# Strategies to eliminate native T cell receptors for adoptive T cell therapies

**DOI:** 10.1016/j.ymthe.2026.01.017

**Published:** 2026-01-20

**Authors:** Donovan Flumens, Diana Campillo-Davo, Florian Van Oers, Philip Anthony Gilbert Shaw, Eva Lion

**Affiliations:** 1 Laboratory of Experimental Hematology, Vaccine & Infectious Disease Institute (VAXINFECTIO), Faculty of Medicine and Health Sciences, University of Antwerp, Antwerp, Belgium; 2 Division of Hematology, Antwerp University Hospital, Edegem, Belgium; 3 Center for Cell Therapy & Regenerative Medicine, Antwerp University Hospital, Edegem, Belgium

**Keywords:** T cell receptor, TCR, T cell engineering, TCR elimination, adoptive T cell therapy, gene engineering

## Abstract

Adoptive T cell therapy has revolutionized modern medicine by harnessing and engineering T cells to selectively target disease-specific antigens. While scientists have developed numerous innovative methods to enhance T cell therapies, strategies to eliminate the native T cell receptor (TCR) have particularly accelerated the field. In TCR T cell therapy, mispairing between native and introduced TCR chains and competition for CD3 binding can compromise therapeutic efficacy and safety. In the context of allogeneic T cell therapies, residual TCR expression can lead to graft-versus-host disease, a life-threatening complication. Effective elimination of native TCRs is therefore essential for improving both safety and efficacy of adoptive T cell therapies. Multiple strategies targeting the TCR at the genomic, transcriptomic, or proteomic level have been developed to achieve effective TCR disruption, each characterized by its own advantages and limitations. This review provides a comprehensive overview of current strategies to eliminate the native TCR in human T cells, highlighting the strengths and weaknesses of each method.

## Graphical abstract

**Figure:**
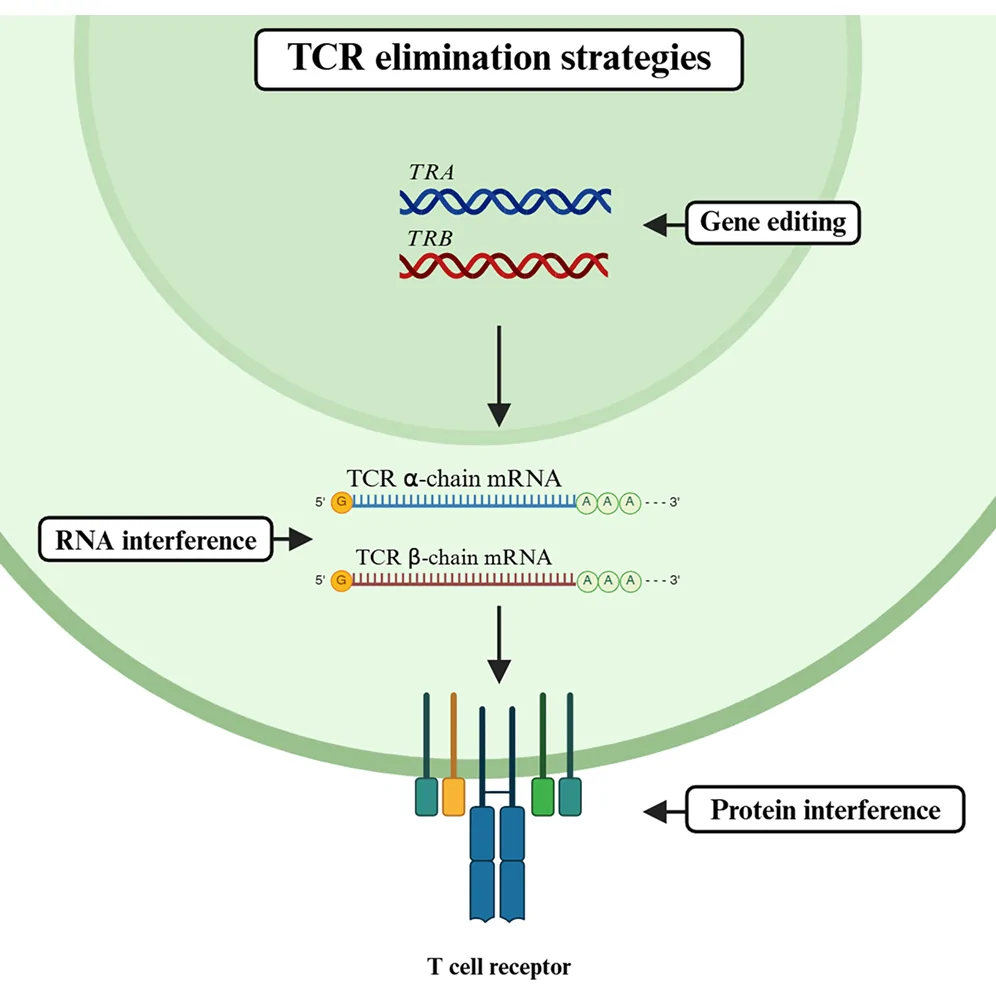

## Teaser

Eliminating native T cell receptors (TCRs) is essential to enhance the safety and efficacy of adoptive T cell therapies by preventing TCR mispairing in TCR T cell therapies and graft-versus-host disease in allogeneic settings. Strategies targeting the TCR at genomic, transcriptomic, and proteomic levels offer distinct advantages and limitations.

## Introduction

T cells are characterized by the presence of T cell receptors (TCRs) on their surface. These molecular sensors recognize peptides presented on major histocompatibility complex (MHC) molecules, triggering a signaling cascade that leads to a targeted T cell response to eradicate unwanted cells, including cancer cells.^1^ Unfortunately, T cells often fail to respond to malignant cells, facilitating immune evasion and cancer progression. To overcome this, T cells can be redirected using tumor antigen-specific TCRs^2^ or artificial chimeric antigen receptors (CARs).^3^ The intrinsic ability of T cells to target diseased cells and their suitability for genetic modification have made these cells attractive candidates for the development of many adoptive cell therapies, a strategy that has revolutionized today’s cancer treatment. Adoptive T cell therapy has rapidly advanced since the early approaches using *ex vivo*-expanded autologous tumor-infiltrating lymphocytes.^4^ In recent years, it has become increasingly easy to genetically modify patient-derived T cells, generate allogeneic off-the-shelf T cell therapies,^5^ and even edit T cells *in vivo*.^6^

While scientists have devised many clever ways to enhance T cell therapies into precise and efficient treatments, strategies to eliminate the native TCRs have particularly accelerated the field. Disruption of the native TCR plays a key role in T cell engineering to avoid mispairing between the native and introduced TCRs, thereby maximizing the specificity and safety of the T cell therapy.^7^ In addition, TCR deletion is essential in the development of allogeneic T cell therapies, preventing graft-versus-host disease (GvHD).^8^^,^^9^ Elimination of the native TCR can be achieved by targeting it at the genomic, transcriptomic, or proteomic level. At the genomic level, gene-editing tools based on programmable nucleases can disrupt TCR gene expression,^10^^,^^11^ while transcriptomic approaches involve RNA interference (RNAi) to silence *TCR* messenger RNA (mRNA).^12^ At the proteomic level, strategies such as antibody-mediated intracellular retention^13^ or protein degradation techniques can be used to prevent TCR surface expression.^14^ In this review, we provide a comprehensive overview of the various engineering platforms developed to eliminate the native TCR of human T cells, highlighting the strengths and weaknesses of each elimination strategy.

### Structure of the TCR-CD3 complex

The TCR is a membrane-anchored heterodimer and a member of the immunoglobulin superfamily of cell surface proteins. In humans, most T cells express a TCR composed of heavily variable and disulfide-linked α and β chains, whereas a minority of T cells express a γδ TCR.^15^ Both αβ and γδ TCRs share a similar architecture, comprising an extracellular domain, a transmembrane domain, and a short intracellular tail.^1^^,^^16^ The extracellular domain of each TCR chain is divided into a membrane-distal variable (V) region and a membrane-proximal constant (C) region.^1^^,^^17^ The V-regions of both the TCR α chain and β chain contain three highly variable complementary determining regions that form an antigen-binding site unique to each TCR. The C-region of the TCR consists of short connecting sequences where a cysteine residue forms an interchain disulfide bond between the TCR α chain and β chain.^18^ The extracellular C-region is followed by a transmembrane domain, which has an essential role during TCR assembly and surface expression by non-covalently interacting with the multisubunit CD3 signaling apparatus and the cell membrane.^19^^,^^20^ Intracellularly, the TCR ends in a minimal cytoplasmic tail that lacks intracellular signaling motifs necessary for T cell activation.^1^^,^^21^ The TCR relies on the interaction with different CD3 proteins to propagate the activation signal after recognizing peptide antigens presented by MHC molecules. These CD3 molecules are organized in three different dimers, namely CD3δε and CD3γε heterodimers and CD3ζζ homodimers, and together with the TCR collectively form the TCR-CD3 complex.^22^^,^^23^ Each of these CD3 subunits contains one or more immunoreceptor tyrosine-based activation motifs (ITAMs) in their cytoplasmic tail, which are indispensable for TCR signaling.^24^^,^^25^ The complete TCR signaling pathway leading to T cell activation has been extensively reviewed by Shah et al*.*^23^

The TCR α chain and β chain are encoded by the respective *TRA* and *TRB* loci, which show an organization in gene segments analogous to immunoglobulins.^26^ The *TRA* locus contains V and J (joining) gene segments (Vα and Jα, respectively), whereas the *TRB* locus also contains D gene segments (Vβ, Dβ, and Jβ). Similar to the immunoglobulin loci, complete and highly variable V-regions are formed by V(D)J recombination during early phases of T cell development in the thymus and determine the specificity of the TCR.^26^^,^^27^ These somatic rearrangements are strictly controlled by the coordinate activity of two proteins encoded by the recombinase-activating genes *RAG1* and *RAG2*.^26^^,^^28^ Regarding the C-region, Cα is encoded by *TRAC*, while *TRBC1* or *TRBC2* genes encode Cβ. To form Cβ, T cells express either *TRBC1* or *TRBC2*, which only differ in four amino acids.^29^^,^^30^ After rearrangement, VJα and VDJβ regions are transcribed and spliced to Cα and Cβ, respectively, to form the final TCR α and β chains.

### Benefits of eliminating the native TCR

In TCR-redirected T cell therapies, T cells are genetically engineered to express transgenic antigen-specific TCRs. However, the presence of the native TCR can hinder the expression of the introduced TCR due to TCR mispairing^7^^,^^31^ and competition for CD3 subunits.^32^^,^^33^ Reduced expression of the desired TCR may compromise the efficacy of TCR-engineered T cell therapies.^33^^,^^34^ Moreover, incorrect pairing of native and introduced TCR α and β chains can form novel TCRs with unpredictable and potentially harmful neoreactivities.^35^^,^^36^ These receptors, whose specificity is unknown, did not undergo thymic selection and have been reported to promote alloreactive or autoreactive T cell responses *in vitro*,^35^ even causing lethal reactions in murine models.^35^^,^^36^ Over the years, different strategies have been investigated to address TCR mispairing and prevent unwanted side effects. Initial approaches relied on promoting preferential pairing of the introduced TCR chains and focused on modifying the C-region. As such, C-regions have been replaced by murine C-segments in a process called murinization^37^^,^^38^ or C-regions derived from γδ TCRs.^39^ Moreover, Cα and Cβ regions have been swapped within their respective α and β chains to create C-domain-swapped TCRs.^40^ Others have introduced extra cysteine residues (cysteinization) within the C-region^41^^,^^42^ or other amino acid mutations at the α and β chain interface to improve transgenic TCR pairing.^43^ Additionally, larger structural modifications have been proposed to generate single-chain TCRs^44^^,^^45^ or to incorporate CD3ζ, the key component for maintaining stability of the TCR-CD3 complex, in TCR-CD3 fusions.^46^

Furthermore, native TCR elimination has paved the way for allogeneic T cell therapies.^47^^,^^48^ Allogeneic T cells are usually collected from healthy donors and represent a highly attractive alternative to autologous cell sources.^49^^,^^50^ However, one of the major downsides of using allogeneic T cells in adoptive cell therapy is the significant risk of GvHD.^8^ As GvHD happens when donor-derived T cells react against healthy tissues of the patient, its adverse effects can range from mild symptoms, like rash and diarrhea, to life-threatening organ failure in more severe cases.^9^ Disruption of native TCR expression prevents allogeneic T cells from recognizing or responding to any MHC-presented antigens, including those expressed in healthy tissues, thereby limiting GvHD. Allogeneic T cells also offer solutions to some challenges posed by patient-derived T cells. While the latter face limited availability and patient-to-patient variability in quality and fitness^49^ (factors that impact therapeutic effectiveness, consistency, and clinical outcome^51^), allogeneic T cells offer the advantage of being a readily available off-the-shelf cell supply, ensuring a more standardized and consistent therapeutic product. Moreover, allogeneic T cells are normally not impacted by an immunosuppressive environment nor anticancer treatments. These advantages can significantly enhance treatment options for patients, facilitating timely interventions and potentially improving outcomes,^49^ which are further enhanced by native TCR disruption.

#### Gene editing to knock out native TCR genes

**Figure 1:**
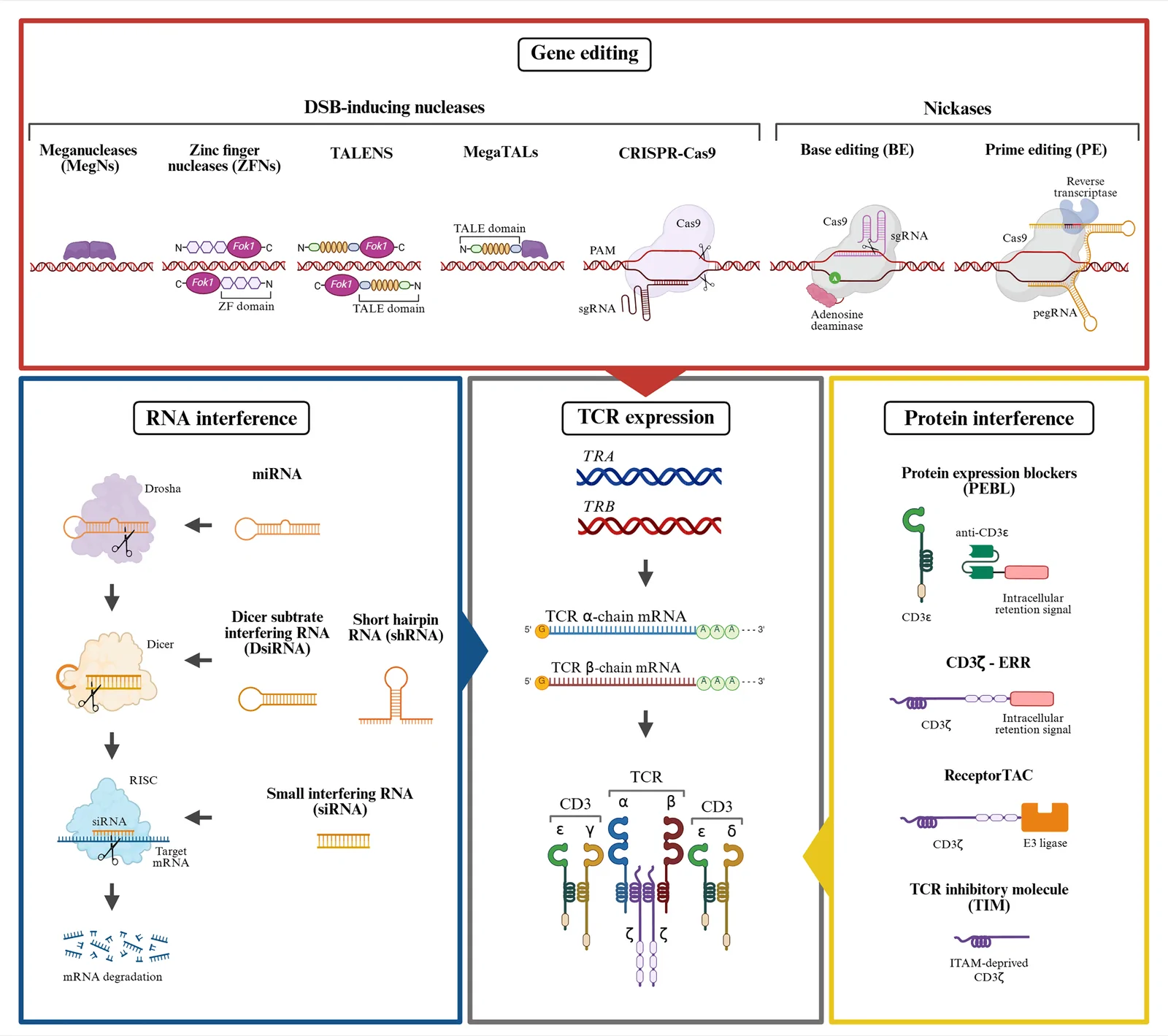
Graphical overview of strategies used to eliminate native TCR expression in human T cells for adoptive T cell therapy The red box highlights nuclease-based gene-editing approaches targeting the TCR at the DNA level. The blue box illustrates various RNAi strategies that suppress TCR expression at the transcriptional or post-transcriptional level, indicating the stage of action within the RNAi pathway. The gray box represents the natural transcription and translation processes of the TCR α chain and β chain. The yellow box shows protein-level interventions that interfere with TCR surface expression and signaling. BE, base editor; Cas9, CRISPR-associated protein 9; CRISPR, clustered regularly interspaced short palindromic repeats; DSB, double-strand break; dsiRNA, Dicer substrate interfering RNA; ERR, endoplasmic reticulum retention signal; ITAM, immunoreceptor tyrosine-based activation motif; MegaTALs, meganuclease-TALE fusion protein; MegNs, meganucleases; miRNA, microRNA; mRNA, messenger RNA; PAM, protospacer adjacent motif; PE, prime editor; PEBL, protein expression blockers; pegRNA, prime editing guide RNA; ReceptorTAC, receptor targeting chimeras; RISC, RNA-induced silencing complex; RNAi, RNA interference; sgRNA, single-guide RNA; shRNA, short hairpin RNA; siRNA, small interfering RNA; TALENs, transcription activator-like effector nucleases; TCR, T cell receptor; TIM, TCR inhibitory molecule; ZF, zinc finger; ZFNs, Zinc-finger nucleases. Figure created in BioRender (https://BioRender.com).

Capable of targeting and modifying precise genomic sequences, gene-editing technologies have been exploited to knock out genes, introduce site-specific mutations, and even insert entire genes, including TCRs. These modifications can be achieved by programmable endonucleases that efficiently create DNA double-strand breaks (DSBs) at specific genomic loci.^52^ These nuclease-induced DSBs drive the activation of cellular DNA repair mechanisms. In the absence of a repair template, DSBs are re-ligated by the non-homologous end joining (NHEJ) pathway, an error-prone repair mechanism that results in the introduction of small insertions or deletions (indels) at the breakpoint.^53^^,^^54^ Indels within a coding exon of the gene can lead to frameshift mutations or premature stop codons, resulting in gene disruption. Alternatively, the high-fidelity homology directed repair (HDR) pathway is harnessed by cells to restore the DSBs.^55^ Compared to NHEJ, HDR repairs the DSBs by using a donor sequence (HDR template), with ends homologous to the targeted genomic site, that can generate complete gene knockins. Although HDR occurs at a low efficiency, it is the desired pathway to generate controlled and precise modifications in the genome, especially for mutations and gene replacement.^55^ Currently, there are four major designer endonuclease platforms with demonstrated potential for gene editing: meganucleases (MegNs), zinc-finger nucleases (ZFNs), transcription activator-like effector nucleases (TALENs), and clustered regularly interspaced short palindromic repeats (CRISPR)-based technologies.^56^ Each platform is defined by its mechanism to recognize and bind the DNA target site (Figure 1) and has distinct advantages and disadvantages (Table 1).

**Table 1:** Native TCR elimination strategies

Elimination strategy	Target level	Target sequence length, nucleotides	Mechanism of action	Efficiency	Transient	Design complexity	Off-target effects	Advantages	Disadvantages
MegNs	DNA	14–44	DNA DSB by homing endonuclease	moderate	no	very high	moderate	• high target specificity • compact size • monomeric nuclease activity	• very hard to reprogram • limited range of target sequences
ZFNs		9–18	DNA DSB by FokI endonuclease	low	no	high	moderate	• compact size • precise targeting	• challenging to design • require dimerization • limited multiplexing capacity
TALENs		12–37	DNA DSB by FokI endonuclease	moderate	no	moderate	moderate	• high target specificity • modular design • broad target range	• require dimerization • complicated vector-based delivery • limited multiplexing capacity
MegaTALs		30–40	DNA DSB by homing endonuclease	high	no	high	moderate	• high target specificity • customizable targeting • monomeric nuclease activity	• challenging to design • limited multiplexing capacity
CRISPR-Cas		20	DNA DSB by sgRNA-guided Cas protein	very high	no	low	moderate	• very efficient • easily designed • easy multiplexing	• genotoxicity • immunogenicity
BE		20	introduction of premature stop codon by single-base conversion	high	no	moderate	low	• no DNA DSBs • precise single-base transitions	• bystander editing • limited base conversion
PE		20	DNA sequence rewriting by reverse transcriptase using pegRNA template	very high	no	high	low	• longer sequence rewriting	• potential unwanted DSBs • delivery can be challenging • more complex to design
miRNA	mRNA	21–23	processed by Drosha and Dicer prior to RISC-mediated cleavage of TCR mRNA	high	yes	low	moderate	• no DNA disruption • endogenous, natural regulation	• difficult to design for single-gene knockdown • lower specificity • rely on integrating vectors
shRNA		21–23	processed by Drosha and Dicer prior to RISC-mediated cleavage of TCR mRNA	moderate	yes	moderate	low	• no DNA disruption • long-term knockdown	• rely on integrating vectors • saturation of RNAi machinery • slower onset than siRNA
dsiRNA		25–30	processed by Dicer prior to RISC-mediated cleavage of TCR mRNA	high	yes	moderate	low	• no DNA disruption • improved silencing • reduced off-target effects	• more complex to synthesize
siRNA		21–23	directly incorporated in RISC to cleave TCR mRNA	moderate	yes	low	low	• no DNA disruption • fast knockdown • easy to design synthetically	• short half-life
PEBL	protein	N/A	intracellular retention by an anti-human CD3ε scFv linked to an ERR	high	yes	low	low	• no DNA disruption	• intracellular accumulation • endoplasmic reticulum stress • rely on integrating vectors
CD3-ERR		N/A	intracellular retention by incorporation of a transgenic CD3ζ linked to an ERR	low	yes	low	low	• no DNA disruption	• intracellular accumulation • endoplasmic reticulum stress • rely on integrating vectors
ReceptorTAC		N/A	targeted degradation by incorporation of CD3ζ linked to E3 ligase	moderate	yes	low	low	• TCR degradation instead of inhibition • no DNA disruption	• rely on integrating vectors • resistance by mutations
TIM		N/A	incorporation of a non-signaling truncated CD3ζ subunit	moderate	yes	low	low	• inhibit TCR signaling • no DNA disruption	• rely on integrating vectors

BE, base editor; CRISPR, clustered regularly interspaced short palindromic repeats; DNA, deoxyribonucleic acid; DSB, double-strand break; dsiRNA, Dicer substrate interfering RNA; ERR, endoplasmic reticulum retention signal; MegaTALs, meganuclease-TALE fusion protein; MegNs, meganucleases; miRNA, microRNA; mRNA, messenger RNA; N/A, not applicable; PE, prime editor; PEBL, protein expression blockers; pegRNA, prime editing guide RNA; RISC, RNA-induced silencing complex; ReceptorTAC, receptor targeting chimeras; RNA, ribonucleic acid; scFv, single-chain variable fragment; shRNA, short hairpin RNA; siRNA, small interfering RNA; TALENs, transcription activator-like effector nucleases; TIM, TCR inhibitory molecule; ZFNs, zinc-finger nucleases.

MegNs, also known as homing endonucleases, are DNA-cutting enzymes found in a variety of bacteria and in eukaryote organelles such as mitochondria.^57^ These naturally occurring endonucleases promote lateral genetic mobility through “homing.” Homing is a process where a mobile sequence is copied into a target allele via site-specific DSBs, enabling the spread of genetic elements within genomes.^58^ MegNs are characterized by their ability to recognize exceptionally long DNA target sequences, typically ranging from 14 to 44 bp. Having long recognition sites endows MegNs with high target specificity and substantially reduces the risk of off-target effects. Furthermore, their relative compact structure facilitates easy delivery into cells.^57^^,^^58^ However, one of the major drawbacks of MegNs is the difficulty in customizing them for new genomic targets. Their DNA-binding and -cleavage domains are structurally interdependent, making it hard to modify one without affecting the other. As a result, the development of custom MegNs is both labor-intensive and expensive, limiting their application as a gene engineering tool.^57^^,^^58^ These challenges are reflected by the fact that, to date, only one study has reported the successful use of MegNs to eliminate the native TCR.^59^ MacLeod et al. developed a single-chain variant of the I-CreI homing endonuclease, called TRC1-2, to target 22 bp within exon 1 of *TRAC*. When introduced into activated T cells, TRC1-2 MegNs achieved over 60% disruption of the native TCR. Building on this strategy, the researchers used an adeno-associated virus (AAV) vector to deliver a donor template encoding a CD19-specific CAR. Combined MegN-mediated TCR knockout and delivery of CD19 CAR transgene enabled targeted integration of CD19 CAR in *TRAC*, with approximately 40% of T cells expressing the CAR with combined surface CD3 suppression.^59^ These off-the-shelf MegN-engineered CD19 CAR-T cells are currently undergoing clinical testing for relapsed/refractory B cell malignancies.^60^

ZFNs are chimeric nucleases consisting of DNA-binding zinc-finger domains linked to the catalytic domain of the type II restriction endonuclease FokI.^61^ Each zinc-finger motif binds to a specific three-base DNA sequence; by combining multiple zinc fingers, ZFNs can be designed to target longer DNA sequences. Typically, the DNA-binding domain of ZFNs is composed of 3–6 zinc fingers, allowing for the recognition of DNA sequences ranging from 9 to 18 bp.^61^^,^^62^ However, it can be challenging to produce zinc fingers that effectively bind to all DNA triplet combinations, limiting the flexibility of the system.^63^ The FokI endonuclease domain introduces a DSB in the DNA but requires the dimerization of two monomeric domains to facilitate DNA cleavage.^64^ As a result, ZFNs must be engineered to target opposite DNA strands, promoting dimerization of FokI. The requirement to bind two proteins enhances the specificity of the ZFN gene-editing tool. Unfortunately, it also makes the design of ZFNs more complex, time consuming, and costly.^63^ Torikai et al. demonstrated that ZFNs targeting either the α or β chains of native TCRs effectively disrupted TCR expression, yielding up to 30% CD3^–^ CAR-T cells when editing *TRAC* and 26% when targeting *TRBC*.^65^ Subsequent magnetic depletion of CD3^+^ cells enabled the enrichment of CD3^–^ CAR-T cells to over 90%. Provasi et al. used a sequential ZFN editing approach to eliminate both TCR chains for the generation of Wilms’ tumor 1 (WT1)-specific TCR T cells with minimal TCR mispairing.^66^ With this protocol, they achieved a knockout efficiency of approximately 19% CD3^–^ cells after two rounds of ZFN exposure, first targeting *TRAC* and then *TRBC*.

TALENs share a comparable structure with ZFNs, consisting of customizable DNA-binding TALEs fused to the catalytic domain of FokI.^67^ These TALEs are naturally occurring DNA-binding proteins secreted by *Xanthomonas* spp. to alter gene expression in host plant cells after infection.^68^ The target specificity of TALEs is determined by arrays of nearly identical amino acid repeats flanked by additional TALE-derived domains. Each TALE typically contains between 12 and 37 repeats, with each repeat comprising 33–35 amino acids and recognizing a single DNA base. By modifying the polymorphic amino acids at positions 12 and 13 in each repeat, TALEs can be engineered to bind all 4 DNA bases and almost any DNA sequence of choice.^69^^,^^70^ The long recognition site and minimal sequence constraints give TALENs a high target specificity; however, their large size and the high repetitive nature of TALE arrays complicates vector-based delivery and makes their design labor-intensive.^71^^,^^72^ Notably, stable expression of designer nucleases is neither required nor desirable, as prolonged nuclease activity increases the risk of off-target effects. Transient delivery approaches, such as mRNA electroporation, address this limitation by enabling efficient genome editing while minimizing nuclease persistence and the associated off-target activity.^72^ After binding to the DNA, the FokI nuclease domain is responsible for introducing a DSB. Since FokI cleaves as a dimer, co-localization of two TALEN monomers is required for nuclease activity.^64^ Several studies have explored TALEN-mediated gene knockout for the elimination of native TCRs in T cells. Poirot et al. generated off-the-shelf CD19-directed CAR-T cells by simultaneously disrupting *TRAC* and *CD52* using TALENs, obtaining on average 54% double-knockout T cells.^47^ These allogeneic CD19 CAR-T cells were then used for treating two infants with B cell acute lymphoblastic leukemia (B-ALL), resulting in molecular remission in both children.^10^ This and several other ongoing clinical trials underscore the clinical potential of TALEN-based genome editing in adoptive T cell therapies.^73^ Moreover, combining multiplex TALEN-mediated ablation of *TRAC* and β2 microglobulin (*B2M*) with AAV-based CAR delivery enables the production of immune-evasive, *TRAC*-inserting universal CAR-T cells.^74^ Additionally, TALENs were used for reprogramming T cells to express an influenza-specific TCR while avoiding TCR mispairing. To achieve this, Berdien et al. designed TALENs targeting both the TCR α chain and β chain, achieving average efficient knockout rates of 58% and 41%, respectively.^75^

To address the individual limitations of MegNs and TALENs, the Scharenberg group developed megaTALs, a hybrid gene-editing tool that combines the sequence targeting flexibility of TALEs with the high cleavage specificity of MegNs.^76^ MegaTALs were achieved by fusing TALE repeat domains to the N terminus of I-OnuI homing endonuclease. Unlike ZFNs and TALENs, megaTALs do not rely on the dimerization of FokI. As a result, efficient gene editing can be achieved by introducing a single, compact, and target-specific molecule into the cell.^76^ Activated T cells treated *in vitro* with megaTALs designed to target the *TRAC* locus showed CD3 disruption rates of approximately 80% as evidenced by the loss of CD3 membrane expression.^77^

A widely known nuclease platform is the CRISPR-Cas system, originally discovered as part of a bacterial defense system against viruses, where it serves to recognize and cut viral DNA.^78^^,^^79^ This system typically consists of two components: a single Cas protein, most commonly Cas9, and a single-guide RNA (sgRNA), which is a fusion of a CRISPR RNA with a *trans*-activating CRISPR RNA.^80^^,^^81^ The sgRNA directs the Cas protein to a specific DNA target sequence of 20 bp, where it cleaves the DNA 3 nt upstream of the protospacer-adjacent motif, generating a DNA DSB. Among all available TCR elimination strategies, CRISPR-Cas9 stands out for its robustness and ease of customization, consistently achieving native TCR knockout efficiencies above 90%.^31^^,^^82^ This further translated into numerous clinical trials using adoptive T cells in which the native TCR is eliminated using this technology.^73^ The two-component nature of the CRISPR-Cas system makes multiplex gene engineering effortless. A Cas protein can be guided to different loci by simultaneously introducing multiple sgRNAs. CRISPR-Cas9 has been successfully used for multiplex gene editing in universal CAR-T cells, knocking out not only the native TCR but also *B2M* and immune checkpoints such as programmed cell death protein 1.^48^^,^^83^ Like other nucleases, CRISPR-Cas has been used for targeted transgene knockin at the TCR gene loci.^84^^,^^85^^,^^86^ Taking this further, Schober et al. demonstrated orthotopic TCR replacement, where the native *TRAC* and *TRBC* loci were precisely inserted with transgenic TCR α and β chain sequences, respectively.^11^^,^^87^ More recently, in-frame CRISPR knockin of a CD3ζ-deprived CAR at the CD3ζ locus resulted in over 30% of CD3^–^ CAR^+^ T cells.^88^ This approach was shown to hijack the TCR signaling complex while disrupting native TCR expression, an effect not observed when the CAR is targeted to the CD3ε locus.^89^ Nonetheless, CRISPR comes with notable drawbacks, including its higher risk of off-target edits, as sgRNAs can tolerate small mismatches in DNA sequences.^90^ Since its development as a DNA-editing tool, extensive efforts have been made to enhance CRISPR-Cas efficiency and reduce off-target effects by optimizing sgRNA design,^91^ engineering better Cas proteins,^92^^,^^93^ exploring alternative Cas nucleases such as Cas12 variants,^93^^,^^94^ and creating Cas fusion proteins with other nuclease domains, as exemplified with Cas-CLOVER.^95^

Base editors (BEs) and prime editors (PEs) are some of the latest additions to the CRISPR genome-engineering toolkit and were developed to overcome the limitations of DSB-based editing. BEs are CRISPR-derived systems that allow precise single-nucleotide conversions without inducing DSBs.^96^ These technologies combine a catalytically impaired Cas nickase with a deaminase enzyme. Guided by a gRNA, the nickase generates a single-strand break at the target site, creating a small DNA window in which the deaminase converts a specific base. This change is then permanently incorporated by the replication or repair machinery of the cell, all without introducing DSBs. To date, two main classes of BEs have been described: cytosine BEs, which mediate cytosine-guanine to thymine-adenine transitions,^96^ and adenine BEs, which convert adenine-thymine to guanine-cytosine.^97^ These BEs can be used to inactivate TCR genes by introducing nonsense mutations that lead to premature stop codons, resulting in a more than 90% loss of TCR protein expression.^98^^,^^99^ Base-edited allogeneic CD7-directed CAR-T cells are currently being investigated in both adult^100^ and pediatric patients^101^ with relapsed/refractory T cell ALL. PEs are donor DNA template-free genome-editing tools capable of rewriting DNA sequences beyond single-nucleotide transitions.^102^ Instead of relying on a DNA-modifying enzyme, PEs consist of a Cas9 nickase fused to a reverse transcriptase and are guided by a prime editing guide RNA (pegRNA). This pegRNA not only guides the PE to the target DNA sequence but it also contains a primer-binding site and serves as the template for introducing the desired genetic modification. Once the PE binds to the target DNA, the Cas9 nickase introduces a site-specific single-strand break, generating a single-stranded DNA flap that serves as a substrate for reverse transcriptase-mediated editing. A primer-binding site encoded within the pegRNA hybridizes to its complementary sequence on the exposed DNA strand, allowing the reverse transcriptase to copy the desired template sequence from the pegRNA directly into the genomic DNA.^102^ Importantly, the Cas9 nickase variant H840A, originally used in PEs, has been reported to occasionally induce unintended DSBs, leading to unwanted indels.^103^ PEs have been successfully employed for site-specific integration of a CD19 CAR into the *TRAC* locus, generating allogeneic CD19 CAR-T cells with over 80% CAR^+^-T cells.^104^^,^^105^ These engineered T cells demonstrated potent antitumor activity in tumor-bearing mouse models.

#### Interfering with TCR expression on a transcriptional level

RNAi, alternatively known as RNA silencing, is a post-transcriptional regulatory mechanism that employs small RNA molecules as guides for sequence-specific downregulation of gene expression.^106^ The RNAi process starts with the cleavage of double-stranded RNA by the cytoplasmic RNase enzyme Dicer, producing small interfering RNAs (siRNAs) of approximately 21–23 bp in length. These siRNA fragments are then loaded into a multiprotein RNA-induced silencing complex (RISC). After strand separation, RISC is directed to complementary mRNA targets, resulting in mRNA cleavage and translational repression.^106^^,^^107^ These endogenous RNAi pathways can be artificially harnessed for gene engineering by introducing synthetic small RNAs specifically designed for a gene of interest.^34^^,^^108^ The attractiveness of RNAi arises from its high specificity and potent inhibitory activity and the fact that it does not cause permanent gene disruption. However, the effects of RNAi are short-lived and demand constitutive expression of the siRNA to achieve sustained TCR knockdown. An integrating vector system, such as viral vectors or transposons can be used to deliver DNA encoding targeted RNAi molecules into T cells. While these methods can provide stable expression, they also introduce safety concerns such as the risk of insertional mutagenesis.^109^

There are several ways to initiate RNAi (Figure 1), which differ based on their entry point in the RNAi pathway (Table 1). MicroRNAs (miRNAs) are usually introduced as primary miRNAs and are first processed in the nucleus into precursor miRNAs by the ribonuclease Drosha. Next, pre-miRNAs are exported to the cytoplasm, where they are further cleaved by Dicer into mature double-stranded miRNAs, which are incorporated into the RISC.^110^ To suppress native TCR expression with miRNAs, Okamoto et al. designed a retroviral construct encoding clusters of primary miRNAs targeting the native TCR combined with a siRNA-resistant antigen-specific transgenic TCR.^34^^,^^111^ This approach allowed simultaneous RNAi-mediated knockdown of the native TCR and expression of the transgene. In a separate study, miRNAs were used to achieve over 90% reduction in native TCR expression, effectively mitigating TCR gene transfer-induced GvHD caused by TCR mispairing in a mouse model.^112^ Short hairpin RNAs (shRNAs) are designed to closely mimic the structure of these miRNAs.^113^ Like miRNAs, shRNAs form a characteristic hairpin loop consisting of two complementary 19- to 22-nt sequences joined by a short loop of 4–11 nt. This structural resemblance allows shRNAs to engage the RNAi pathway in a manner similar to that of miRNAs. However, unlike miRNAs, shRNAs bypass the need for Drosha-mediated processing and are directly recognized and cleaved by Dicer for faster processing and more rapid induction of gene silencing.^113^ When delivered via viral vectors, shRNAs can integrate into the host genome, enabling stable and long-term gene knockdown. Compared with miRNAs, shRNAs targeting the native TCR were less effective overall but still achieved substantial knockdown, with shRNAs reducing native TCR α and β expression to 10% and 60%, respectively, and miRNAs reducing both to approximately 20% of the levels observed in control T cells.^34^ Nonetheless, shRNAs targeting the TCR are being clinically validated in B cell maturation antigen-directed - cell therapy for the treatment of relapsed or refractory multiple myeloma.^114^ An alternative approach to knock down the native TCR is the direct delivery of synthetic Dicer-substrate interfering RNA (DsiRNA) of 25–30 bp in length into the cytosol.^108^ As the name suggests, these longer siRNA duplexes are optimized for Dicer processing before incorporation into RISC, offering enhanced potency, efficiency, and prolonged silencing compared with conventional siRNAs. Our research group demonstrated that both TCR chains can be efficiently and simultaneously downregulated by electroporation of DsiRNAs recognizing *TRAC* and *TRBC* transcripts, resulting in more than a 6-fold decrease in *TRAC* and over a 3-fold reduction in *TRBC* mRNA levels compared with control T cells.^12^ Alternatively, siRNAs are also delivered directly into T cells, enabling immediate incorporation into the RISC complex and rapid TCR gene silencing, reducing native TCR α chain and β chain RNA expression to 54% and 39%, respectively, compared with control cells.^34^ Importantly, when RNAi strategies are combined with the introduction of a transgenic TCR, codon optimization of the transgenic TCR sequence is required to protect it from RNAi-mediated downregulation.^12^^,^^34^^,^^112^

### Interfering with TCR surface expression and signaling

Further expanding the arsenal of tools to eliminate the native TCR (Figure 1), several technologies have been established to regulate TCR surface expression or downstream TCR signaling pathways (Table 1). Kamiya et al. developed a method based on the requirement that the TCR-CD3 complex must be fully assembled before it can be transported to the T cell membrane.^13^ To achieve simple yet effective blockade of TCR surface expression, they designed protein expression blockers (PEBLs), which consist of an antibody-derived single-chain variable fragment (scFv) specific for CD3ε, fused to an endoplasmic reticulum retention (ERR) signal. Intracellular colocalization of CD3ε using these PEBLs effectively blocked its transport to the membrane and prevented the formation of TCR-CD3 complexes. As a result, surface expression of the TCR was reduced from approximately 96% to –25%.^13^ A PEBL directed against the native TCR is currently being investigated in an allogeneic CD19 CAR T cell therapy trial for the treatment of B-ALL.^115^ A similar strategy was employed by Ebrahimiyan et al. to generate allogeneic CD19 CAR-T cells by introducing synthetic CD3 subunits containing an ERR signal via viral transduction.^116^ These CD3-ERR subunits integrated into the TCR-CD3 complex within the ER, thereby preventing the fully formed TCR from reaching the cell surface. Among the engineered subunits, CD3ζ fused with an ERR signal (CD3ζ-ERR) exhibited the most effective TCR downregulation, resulting in an average TCR knockdown efficiency of 34%.^116^

A parallel method to regulate TCR expression at the protein level was demonstrated by Harris et al. and their chimeric E3 fusion proteins called receptor targeting chimeras (ReceptorTAC).^14^ To generate non-alloreactive claudin18.2-specific CAR-T cells, targeted degradation of the native TCR complex was facilitated by direct fusion of E3 ligase domains to the transmembrane domain of CD3ζ. Comparison of several E3 ligase domains revealed that membrane-bound E3 ubiquitin ligases outperform cytosolic variants in downregulating the native TCR, possibly because membrane-bound ligases might act more directly on surface components. A ReceptorTAC based on the intracellular E3 ligase domain of genes related to anergy in lymphocytes caused up to a 50%–60% reduction in TCR expression and strongly attenuated allogeneic T cell reactivity *in vitro*.^14^

Celyad Oncology developed, in addition to regulating TCR protein expression, a TCR inhibitory molecule (TIM) to interfere with TCR signaling pathways.^117^ TIM is a shortened form of human CD3ζ truncated before the first ITAM sequence and therefore functions as a non-signaling subunit. Although previous studies demonstrated that disruption of ITAM sequences alone is insufficient to terminate TCR signaling,^118^ introduction of TIM into T cells led to its incorporation into the TCR-CD3 complex and subsequent blunted TCR responses. No GvHD responses were observed in immunodeficient mice treated with human TIM T cells, indicating the potential favorable safety profile of this technology. This CD3ζ truncation strategy is being used in a clinical trial with non-gene-edited allogeneic natural killer group 2D (NKG2D)-directed CAR-T cells (NCT03692429).^117^^,^^119^

These protein-level interventions focus primarily on engineering or targeting CD3 subunits, in particular CD3ζ, to inhibit expression or signaling of the native TCR. These systems are relatively simple to design; however, their transient nature poses a significant limitation for long-term therapeutic applications. Similar to RNAi strategies, achieving sustainable expression typically requires genomic integration into the host genome.

#### Risks associated with TCR elimination strategies

The elimination of native TCRs, although intended to improve adoptive T cell therapies, is not without risks (Table 2). In TCR-engineered T cell products, incomplete or single-chain disruption of the native TCR can lead to increased mispairing with the transgenic TCR—for example, when the native TCR α chain has no pairing option other than the transgenic TCR β chain.^11^ Therefore, multiplexed editing of both the native α and β chains is required to guarantee exclusive expression of the transgenic TCR. Incomplete native TCR elimination can also pose considerable challenges in allogeneic T cell therapies, where TCR disruption is intended to prevent GvHD. A report described the development of GvHD in two pediatric B cell precursor ALL patients treated with allogeneic TALEN-engineered TCR-disrupted CD19 CAR-T cells.^10^ Despite selection for TCR negativity by depleting TCRα/β^+^ cells, residual TCR^+^ CD19 CAR-T cells were detected and expanded *in vivo*, causing GvHD in these patients.

**Table 2:** Development stage and safety issues of TCR elimination strategies

Elimination strategy	*In vitro*	*In vivo* (mouse models)	*In vivo* (clinical trials)	Relevant references	Safety issues related to TCR elimination strategy
MegNs	✓	✓	✓	MacLeod et al.^59^; Shah et al.^60^	off-target effects: • undesired mutations in unintended genomic locations, potentially leading to tumorigenesis on-target effects: • structural chromosomal abnormalities, including local DNA rearrangements, DNA deletions, chromothripsis, chromosomal truncations, and aneuploidy
ZFNs	✓	✓	–	Torikai et al.^65^; Provasi et al.^66^	
TALENs	✓	✓	✓	Qasim et al.^10^; Georgiadis et al.^73^; Jo et al.^74^	
MegaTALs	✓	–	–	Osborn et al.^77^	
CRISPR-Cas	✓	✓	✓	Morton et al.^31^; Georgiadis et al.^73^	
BE	✓	✓	✓	Webber et al.^98^; Diorio et al.^100^; Chiesa et al.^101^	off-target effects: • undesired mutations in unintended genomic locations, potentially leading to tumorigenesis
PE	✓	✓	–	Pomeroy et al.^105^	
miRNA	✓	✓	–	Okamoto et al.^34^; Bunse et al.^112^	• off-target effects causing sequence-specific silencing of unintended genes • activation of the tumor suppressor p53 pathway independently of their intended gene targets • saturation of the endogenous RNAi machinery, which can lead to competition with native RNAs, disrupting normal gene regulation • RNAi molecules can trigger innate immunity via TLRs, inducing pro-inflammatory cytokines such as IFNs and TNF-α
shRNA	✓	✓	✓	Okamoto et al.^34^; Al-Homsi et al.^114^	
dsiRNA	✓	–	–	Campillo-Davo et al.^12^	
siRNA	✓	–	–	Okamoto et al.^34^	
PEBL	✓	✓	✓	Kamiya et al.^13^; Hu et al.^115^	• non-human elements (e.g., murine sequences) can trigger immune reactions • prevention of membrane trafficking of native TCRs may lead to intracellular TCR accumulation, potentially causing endoplasmic reticulum stress
CD3-ERR	✓	–	–	Ebrahimiyan et al.^116^	
ReceptorTAC	✓	–	–	Harris et al.^14^	
TIM	✓	✓	✓	Michaux et al.^117^; Prenen et al.^119^	

BE, base editor; DNA, deoxyribonucleic acid; dsiRNA, Dicer substrate interfering RNA; ERR, endoplasmic reticulum retention signal; IFNs, interferons; MegaTALs, meganuclease-TALE fusion proteins; MegNs, meganucleases; miRNA, microRNA; PE, prime editor; PEBL, protein expression blockers; ReceptorTAC, receptor targeting chimeras; RNA, Ribonucleic acid; RNAi, RNA interference; shRNA, short hairpin RNA; siRNA, small interfering RNA; TALENs, transcription activator-like effector nucleases; TIM, TCR inhibitory molecule; TLR, Toll-like receptors; TNF-α, tumor necrosis factor α; ZFNs, zinc-finger nucleases.

Complete knockout of the TCR genes using one of the aforementioned genome-editing nucleases seems the best choice in most scenarios. However, none of these nuclease platforms are foolproof, as all have demonstrated instances of unforeseen off-target and on-target genotoxicities in *in vitro* experiments.^90^^,^^120^ One of the most prominent concerns associated with programmable nucleases is undesired mutagenesis, where off-target edits in unintended genomic locations can increase the risk to hazardous cellular consequences.^121^^,^^122^^,^^123^ Fortunately, recent modifications to these nucleases have significantly enhanced their specificity, thereby reducing their off-target recognition.^93^^,^^124^ In addition to off-target effects, it has recently been reported that on-target gene-editing-mediated DSBs can also give rise to potential detrimental consequences. On-target gene editing has been linked to a spectrum of structural chromosomal abnormalities, including local DNA rearrangements and deletions,^125^ as well as more severe events such as chromothripsis,^120^ chromosomal truncations,^126^ and aneuploidy.^126^^,^^127^ Moreover, human clinical trials have shown that multiplexing programmable nucleases to generate multiple on-target DSBs can lead to DNA rearrangements and chromosomal translocations that persist at low levels for months to years.^128^ Encouragingly, these abnormalities have not been associated with any clinical consequences so far^128^ and can be mitigated through minor adjustments to their genome-editing protocols.^129^ Alternatively, non-DSB genome-editing approaches, such as base editing, provide a stable engineering strategy for TCR elimination that avoids the side effects associated with DSB formation.^98^^,^^99^^,^^130^

Although DNA-sparing approaches avoid the genotoxicity associated with genomic editing, they present their own set of risks. RNAi strategies, such as genome-editing methods, are susceptible to off-target effects. RNAi molecules can unintentionally bind to and silence non-target transcripts due to partial sequence complementarity, leading to sequence-specific rather than target-specific side effects and potentially disrupting normal cellular functions.^131^ It has been shown that siRNAs induce unexpected changes in protein levels within the p53 pathway, independently of on-target gene silencing.^132^ In addition, high levels of exogenous RNAi constructs can saturate the endogenous RNAi machinery by overwhelming components such as Dicer and RISC, thereby competing with endogenous RNAs and disrupting normal gene expression.^133^ Another adverse effect of exogenous RNAi arises from its immunostimulatory activity.^134^^,^^135^ Introduction of foreign RNAi molecules can activate innate immune responses through Toll-like receptors, leading to the production of pro-inflammatory cytokines, such as interferons and tumor necrosis factor α. PEBLs, TIMs, and CD3-ERR, however, are less likely to cause adverse effects due to their protein interaction-based mechanisms. Still, when they are constructed using non-human protein sequences, they can also trigger humoral immune responses similar to those observed against murinized transgenic TCRs.^136^ For example, the scFv used in PEBLs targeting the CD3ε subunit was derived from a murine antibody.^13^ Humanizing, the process of modifying non-human antibody sequences to more closely resemble human antibodies, can reduce the immunogenicity of the scFv used in PEBLs.^137^ An additional concern related to methods preventing membrane trafficking of the native TCR is the potential intracellular accumulation of TCRs, which may lead to endoplasmic reticulum stress.^138^ Nevertheless, it is important to recognize that, as shown here, each technology has its own benefits and drawbacks, but the relevance of these risks depends largely on the final application.

#### Benefits of preserving the native TCR

While elimination of native TCRs has become a common practice in the development of adoptive T cell therapies, deliberate preservation of a functional native TCR might be desirable in some defined strategies. This has been clearly demonstrated by the graft-versus-leukemia (GvL) effect observed after human leukocyte antigen -matched donor lymphocyte infusions following allogeneic stem cell transplantation in leukemia patients.^139^^,^^140^ In GvL cases, donor T cells recognize leukemia-specific minor histocompatibility antigens and leukemia-associated proteins to eradicate residual leukemic cells, reducing the risk of relapse.^141^ However, this beneficial GvL effect is inherently linked to the risk of GvHD, as donor T cells may also recognize and attack healthy host tissues. Besides this antitumor effect, preserving the native TCR can promote *in vivo* T cell persistence. As demonstrated in *in vivo* mouse experiments by Labrecque et al., T cells lacking a TCR gradually decay due to the absence of essential TCR-MHC-peptide interactions.^142^ These findings were further supported by Stenger et al., who reported that retaining the native TCR in allogeneic CD19 CAR-T cells enhanced T cell persistence and prolonged disease control in mice, albeit at the cost of increased alloreactivity.^143^ By selecting virus-specific T cells for the development of TCR- and CAR-engineered T cell therapies, this alloreactivity can be minimized, as these cells have a safer profile due to their known TCR specificity against disease-causing viruses.^144^^,^^145^ In addition, preservation of the native TCR in these virus-specific T cells provides an *in vivo* vaccination-like effect, driven by latent viral infections, contributing to the persistence of the TCR- or CAR-engineered T cells.^145^^,^^146^^,^^147^ A clinical trial from 2008 demonstrated that Epstein-Barr virus (EBV)-specific T cells engineered to express a GD-2-specific CAR persist longer in neuroblastoma patients than GD-2 CAR-T cells lacking an EBV-specific TCR, highlighting the advantage of native TCR-mediated activation in promoting T cell persistence.^146^ Similarly, Lapteva et al. used multivirus-specific T cells, targeting EBV, cytomegalovirus, and adenovirus, to generate CD19 CAR-T cells for the treatment of B-ALL and showed that TCR stimulation enhances the expansion and function of the CAR-T cells.^147^

## Conclusions and future perspectives

The field of adoptive T cell therapy continues to advance, with the development of allogeneic off-the-shelf T cell products and *in vivo* T cell engineering as promising paths forward. As these innovations progress, selecting the right TCR elimination strategy and knowing when and if it is required become increasingly important. In TCR-engineered T cell therapies, disrupting the native TCR is necessary to prevent TCR mispairing and competition for CD3 molecules required to form the TCR-CD3 complex. Similarly, in the development of allogeneic T cell therapies, complete and durable elimination of the native TCR is crucial to prevent GvHD, a goal that can be precisely achieved using programmable nucleases targeting the TCR constant regions. These gene-editing tools not only eliminate the native TCR but can also precisely replace it with a transgenic CAR or TCR by leveraging the HDR machinery of the cell. However, genomic editing carries the risk of on-target and off-target side effects, raising concerns about safety. As a result, there is growing interest in DNA-sparing modalities that minimize genomic disruption while maintaining efficacy. Whereas these alternatives mitigate the risks associated with nucleases, they often result in incomplete TCR elimination and, therefore, typically need to rely on integrating vectors for constitutive TCR downregulation, coming back to the risk of mutagenesis. In certain contexts, preserving the native TCR can even be beneficial, as it may enhance the persistence and longevity of the engineered T cell product.

Ultimately, selecting an elimination strategy requires balancing trade-offs, with the optimal method determined by the level of risk acceptable for a given therapeutic product and disease. To ensure clinical applicability, these approaches must be integrated into scalable, Good Manufacturing Practice-compliant protocols with robust quality control measures to verify the accuracy of TCR elimination and the minimization of risks concerning treatment-related tumorigenesis, as observed in a few cases in the context of CAR-T therapy.^148^ Regulatory compliance and comprehensive safety monitoring are essential throughout the production and clinical application process, to avoid the development of (new) adverse clinical conditions. Further clinical translation and in-human testing will be necessary to uncover the remaining gaps and limitations of each technology.

## Acknowledgments

D.F. was funded by an Emmanuel van der Schueren starters grant from Stand Up to Cancer (Kom op tegen Kanker, Belgium; Grant MulTplex), followed by a DOCPRO4 PhD grant of the Special Research Fund (BOF; Grant MulTplex) of the 10.13039/501100007660University of Antwerp. D.C.-D. holds a Junior postdoctoral fellowship from the Research Foundation-Flanders (FWO [Fonds Wetenschappelijk Onderzoek]; grant no. 12B2W24N). F.V.O. was supported by an FWO senior clinical investigator fellowship obtained by Dr. Sébastien Anguille (grant no. 1806225N). P.A.G.S. received funding from the Special Research Fund (BOF) of the 10.13039/501100007660University of Antwerp via a DOCPRO4 PhD grant (grant no. 44239). E.L. was supported by the Methusalem Funding Program from the 10.13039/501100007660University of Antwerp.

## Author contributions

D.F. conceived this review, reviewed the literature, and wrote the original manuscript. D.C.-D., F.V.O., P.A.G.S., and E.L. edited and critically revised the manuscript, contributing to its final version. D.F. prepared the figures and tables. D.C.-D. and E.L. supervised the work. All authors read and approved the final manuscript.

## Declaration of interests

The authors declare no competing interests.
